# Processable high internal phase Pickering emulsions using depletion attraction

**DOI:** 10.1038/ncomms14305

**Published:** 2017-02-01

**Authors:** KyuHan Kim, Subeen Kim, Jiheun Ryu, Jiyoon Jeon, Se Gyu Jang, Hyunjun Kim, Dae-Gab Gweon, Won Bin Im, Yosep Han, Hyunjung Kim, Siyoung Q. Choi

**Affiliations:** 1 Department of Chemical and Biomolecular engineering and KINC, KAIST, Daejeon 305-701, Korea; 2 Department of Mechanical Engineering, KAIST, Daejeon 305-701, Korea; 3 Applied Quantum Composites Research Center, Korea Institute of Science Technology (KIST), Jeonbuk 565-905, Korea; 4 School of Materials Science and Engineering and Optoelectronics Convergence Research Center, Chonnam National University, Gwangju 61186, South Korea; 5 Department of Mineral Resources and Energy Engineering, Chonbuk National University, Jeonju-si, Jeollabuk-do, 561-756, Korea

## Abstract

High internal phase emulsions have been widely used as templates for various porous materials, but special strategies are required to form, in particular, particle-covered ones that have been more difficult to obtain. Here, we report a versatile strategy to produce a stable high internal phase Pickering emulsion by exploiting a depletion interaction between an emulsion droplet and a particle using water-soluble polymers as a depletant. This attractive interaction facilitating the adsorption of particles onto the droplet interface and simultaneously suppressing desorption once adsorbed. This technique can be universally applied to nearly any kind of particle to stabilize an interface with the help of various non- or weakly adsorbing polymers as a depletant, which can be solidified to provide porous materials for many applications.

## Abstract (web summary)

The fabrication of emulsion droplets stabilized by solid particles adsorbed on the interface is restricted to delicate interfacial conditions. Here, Kim *et al*. show a general approach to prepare them using the depletion interaction, modified by soluble polymers, between particles and emulsions.

High internal phase emulsions (HIPEs) that have a high volume fraction (*φ*>74%) of dispersed droplets in a low volume fraction of continuous phase^1^ have been attractive for use in a wide range of areas^2^,^3^,^4^,^5^,^6^,^7^,^8^,^9^ because of their interesting features, such as a large surface area per volume of continuous phase^1^,^3^,^4^ and solid-like flow behaviour because of osmotic pressure^10^,^11^. Taking advantage of these features, HIPEs have been used as templates for porous materials^2^,^3^,^4^ used in various applications, such as organic semiconductors^5^, filter membranes^6^, scaffolds for tissue engineering^7^, food products^8^ and drug delivery systems^9^.

High internal phase Pickering emulsions (HIPPEs) stabilized by colloidal particles possess superior stability compared with HIPEs stabilized by low molecular surfactants because of the irreversible adsorption of particles to the interface^12^,^13^. However, it has been thought to be extremely difficult to fabricate HIPPEs because phase inversion occurs at *φ*>0.7 (refs 12, 14, 15), as predicted by theory^14^ and demonstrated in experiments^12^,^15^. This phase inversion could be prevented by the use of particles that are well dispersed in a continuous phase, but they would not adsorb to the interface easily, resulting in difficulty to form even Pickering emulsions. Nevertheless, several groups have succeeded in creating HIPPEs under very limited conditions, either by the delicate surface modification of colloids^16^,^17^,^18^,^19^,^20^,^21^ or by the use of synthetic colloids that possess the appropriate surface properties^22^,^23^,^24^,^25^.

In this article, we exploit the depletion interaction^26^, another type of physical interaction that can produce attraction between a droplet and a hydrophilic particle (Fig. 1a), enhancing particle adsorption to the droplet interface; thus, this attractive interaction could potentially improve the stability of emulsions while the hydrophilic particles would inhibit phase inversion. Furthermore, particle desorption from the interface would be also suppressed by the depletion attraction, thus providing a great stability of HIPPEs. Taking advantages of these, we systematically investigate a role of depletion attraction between a colloidal particle and a liquid droplet to fabricate stable and even processable HIPPEs using various particle-depletant combinations.

## Results

### Enhanced stability of Pickering emulsion with PEG

**Figure 1:**
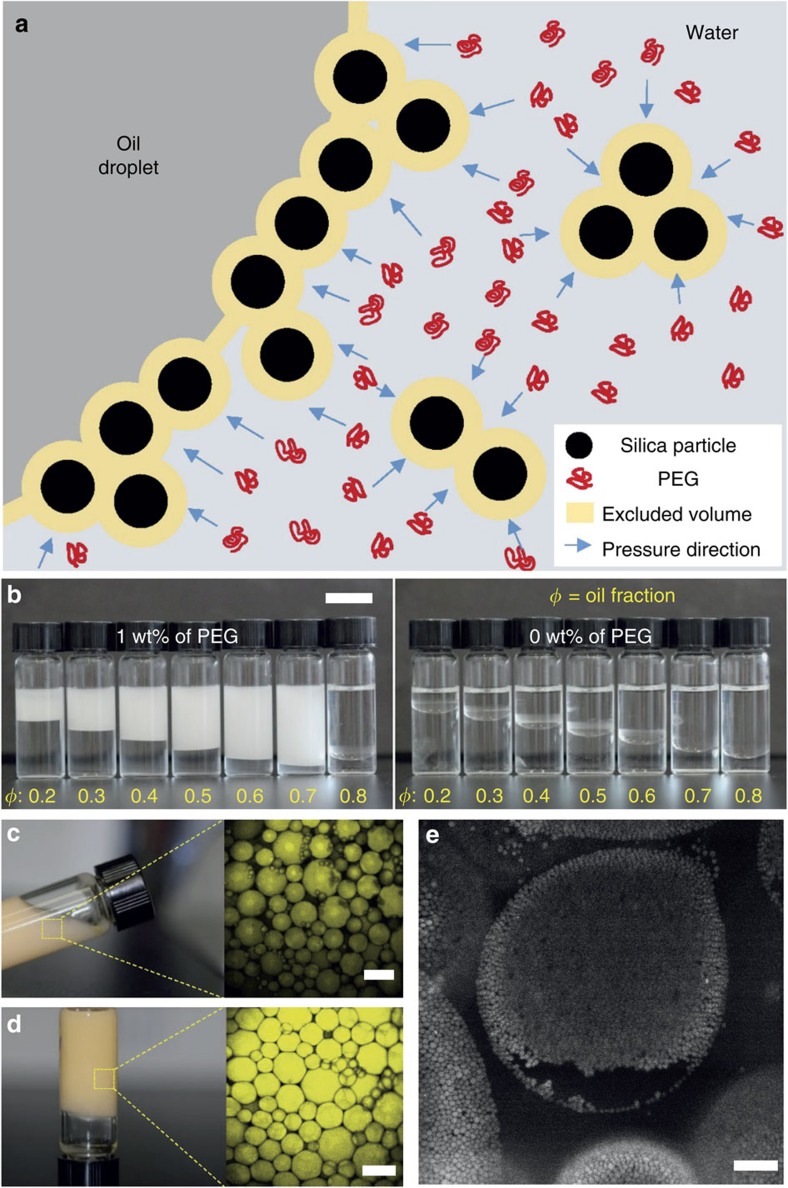
Depletion attraction used for Pickering emulsions. (**a**) Schematic illustration of particle adsorption to the interface by depletion attraction. (**b**) Photographs of compared emulsions (0.18 vol% of 1 μm-silica particles) formed with the presence of PEG (10,000 g mol^−1^, left) and the absence of PEG (right), taken after several hours from voltex emulsification (∼3,000 r.p.m.). Here, *φ* is the oil fraction of the initial oil–water mixture before emulsifying. Scale bar, 1 cm. (**c**) Photograph of *φ*=0.6 emulsion (0.18 vol% silica particles) with oil-soluble fluorescence dyes and its confocal micrograph. Scale bar, 100 μm. (**d**) Photograph of *φ*=0.8 high internal phase Pickering emulsion (0.18 vol% silica particles) and its confocal micrographs. Scale bar, 100 μm. (**e**) Confocal microscope image of fluorescently labelled silica particles (1.08 vol% silica particles) well adsorbed on an oil droplet surface by depletion attraction. Scale bar, 30 μm. All emulsions and HIPPEs (**b**–**e**) are produced by the vortexing method.

We first compare the emulsion stability in the presence/absence of polymers as a depletant^27^,^28^,^29^,^30^. Hexadecane as an oil phase are added to aqueous solutions of hydrophilic silica particles in the presence/absence of polyethylene glycol (PEG), and then oil is broken into smaller droplets by simple vortexing (low-energy emulsification). Oil in water (O/W) emulsion using 0.18 vol% silica particles of 1 μm in diameter, and 1 wt% of PEG, are well stabilized for volume fractions up to *φ*=0.7 (Fig. 1b; Supplementary Fig. 1). They remain unchanged for at least several days. On the other hand, O/W emulsions without polymers or without particles become unstable immediately, leading to complete phase separation (Supplementary Fig. 2). To verify the emulsion stability microscopically, we directly visualize the O/W emulsion with an oil-soluble fluorescence dye using confocal microscopy and find that the size of the oil droplets remained the same over several days (Fig. 1c,d; Supplementary Fig. 3). Using fluorescently labelled silica particles, we also confirm that emulsions are stabilized mainly by adsorbed particles, instead of PEG (Fig. 1e). This suggests that the particle adsorption could be enhanced by depletion attraction.

It is noteworthy that the depletion attraction might be applied between droplets and between the particles, as well as between a droplet and a particle. The depletion attraction between droplets could not work because particles at the droplet interface provide a space between two droplets for PEGs to freely go in and come out, even when two droplets are in contact. For the depletion attraction between particles, if particles are flocculated by the depletion attraction during the emulsifying process, it helps to stabilize emulsions^31^, but no significantly flocculated particles are observed in any of our systems (Fig. 1e; Supplementary Figs 4 and 5). Although particles could form aggregates by the depletion attraction, a large external force for emulsification with the help of the repulsive interaction between silica particles could break particle aggregates into an individual particle. On the other hand, the particle could better remain at the interface against the external force because of the surface tension.

### Enhanced particle adsorption via depletion attraction

**Figure 2:**
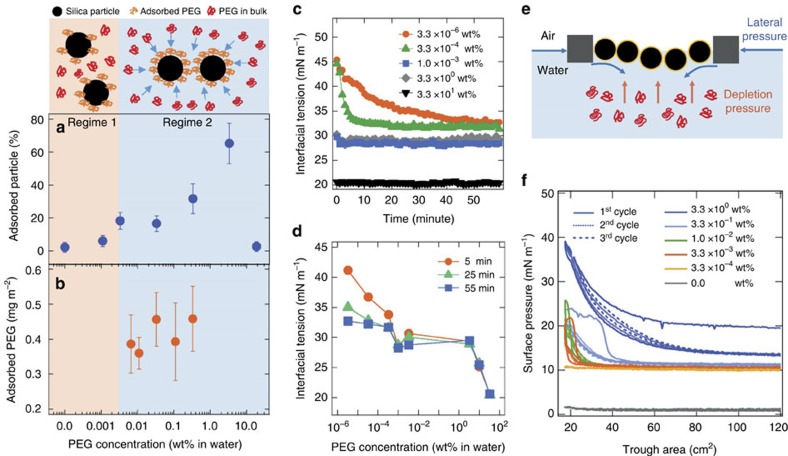
Desorption and adsorption of silica particles. (**a**) Particle fraction adsorbed to the interface by emulsification as a function of PEG concentration. Top: simple schematics for various roles of PEG in bulk with varying concentrations. (**b**) Amount of PEG adsorbed to the silica surface as a function of PEG concentration. Error bars denote s.d. of the value estimated in three different experiments. (**c**) Interfacial tension measurements of an oil–water interface with time by a pendent drop method at each concentration of PEG in a water droplet. (**d**) Interfacial tension with various PEG concentrations at 5, 25 and 55 min, respectively. (**e**) Simple schematic presenting the suppressed desorption of particles from the interface, induced by depletion pressure. (**f**) Langmuir isotherm of solvent-spread silica particles at the air/aqueous solution with various PEG concentrations.

To investigate the role of PEG more quantitatively, we first measure the particle adsorption to the oil–water interface and PEG adsorption to the particle surface (Fig. 2a,b). Starting from nearly zero adsorption without PEG, at low PEG concentrations (<0.0033, wt%, regime 1), particle adsorption to the oil–water interface increases with PEG concentration to ∼20%. High PEG concentrations (0.0033, wt% <*C*_PEG_ <3.3 wt%, regime 2), enhances the particle adsorption dramatically up to ∼70% until *C*_PEG_=3.3 wt%, but adsorption of particles sharply decreases at higher PEG concentrations (Fig. 2a). In regime 1, PEG adsorption to the silica surface increases from 0 mg m^−2^ to ∼0.45 mg m^−2^ (Fig. 2b), and its adsorption to the oil–water interface increases with increasing PEG concentration as well (Fig. 2c,d). These observations suggest that particle adsorption onto the oil–water interface is mainly determined by the amount of PEG adsorbed onto the silica surface, which changes the particle wettability, and the depletion pressure is too weak to enhance the particle adsorption in regime 1.

Above 0.033 wt% of PEG (regime 2), depletion pressure determines the adsorption behaviour of particles (Fig. 2a). PEG adsorption to the silica surface (Fig. 2b) and the oil–water interface (Fig. 2c,d) indicates the amount of adsorbed PEG is already saturated at a much lower concentration, while the depletion pressure in the bulk phase should increase continuously with PEG concentration. The maximum repulsive force per unit area between a silica particle and an oil droplet was previously measured to be ∼10 kPa by AFM (in Derjaguin approximation, an energy per unit area is equivalent to a measured force/particle radius, and the repulsive pressure could be thus computed from the slope of the force/radius—distance graph.)^32^, and our depletion pressure *P*∼*cRT*∼10 kPa for 1 wt% of 10 kDa PEG in an ideal case and *P*∼100 kPa when considering an excluded volume of PEGs^33^,^34^. Furthermore, it can be >100 kPa for deformable interfaces^35^,^36^. Therefore, the depletion attraction could overcome the energy barrier of particle–oil interaction so that particles could reach the interface. These particles are proved to be irreversibly adsorbed to the interface when C_PEG_ is in regime 2, suggesting that capillary force at the three phase contact line holds the particles at the interface (Supplementary Fig. 6).

On the other hand, the adsorption of particles sharply decreases at higher PEG concentrations, ∼10 wt%. As shown in Fig. 2c,d; Supplementary Fig. 7, the interfacial tension decreases suddenly at higher concentration of PEG, ∼10 wt% while the amount of PEG at the oil–water interface stays almost similar even with increasing the PEG concentration from ∼0.001 wt% to ∼5 wt% and ∼0.01 wt% to ∼5 wt% for 10 k PEG and 1.5 k PEG, respectively. It is presumably that a large amount of PEG adsorbed to the interface before adsorption of the particles acts as a polymer brush, thus disturbing the particle adsorption.

### Suppressed particle desorption via depletion attraction

Depletion attraction could also suppress desorption of particles from the interface as well, once particles are adsorbed. As soon as the adsorbed particles escape from the interface, the depletion attraction would place them back to the interface (Fig. 2e). To prove this suppression more quantitatively, we measure the Langmuir isotherms of solvent-spread 1-μm silica particles on an air/aqueous solution with various PEG concentrations (Fig. 2f). In the absence of or with low concentrations of PEG, the surface pressure remains at zero upon the spreading particles. We find that most particles submerge in the bulk water phase. On the other hand, when the same amount of particles is spread on the PEG or dextran solution, far more silica particles is left on the surface such that they could even be observed by eye (Supplementary Figs 8 and 9a). This different particle amount remained at the surface after spreading supports that the depletion attraction plays an important role in forming a more stable particle layer at the air/water interface. The amounts of particles at the interface increases substantially as increasing the concentrations of PEG solutions from 0.0033, wt% to 3.3 wt%, while PEG adsorption to silica particles (Fig. 2) and to interfaces (Supplementary Fig. 9b) remain the same at this concentration regime. Therefore, depletion attraction is likely to be responsible for the suppressed desorption, although other possible mechanisms such as particles bridging by PEGs could help them stay at the interface.

We should note that particles could increase the surface pressure when at least10 times more particles are spread at the air/water interface without PEG, and it could increase the surface pressure even more on compression (Supplementary Fig. 9c). However, a few compression-expansion cycles make a significant difference. After the first compression with large hysteresis that corresponds to large structural reorganization (Supplementary Fig. 8), silica particle layers on the PEG solution show minimal hysteresis, demonstrating that most of the silica particles are left on the interface even for multiple compression–expansion cycles (Fig. 2f). On the other hand, significant amount of silica particles at the air/water interface are desorbed from the interface during the first cycle, thus showing the surface pressure saturation after a few cycles (Supplementary Fig. 9c). We also find from the estimation that the maximum magnitude of desorption force to remove particles held by the surface tension is smaller than the magnitude of depletion force (Supplementary Fig. 10).

These observations with simple estimations strongly suggest that the depletion pressure is the key factor to determine the particle adsorption/desorption behaviours rather than other possible roles of PEG. PEGs adsorbed to the silica surface and the oil–water interface might play a minor role for the adsorption/desorption behaviour^31^,^37^. However, PEG adsorption to the oil–water interface and to the silica surface is almost constant over three orders of magnitude PEG concentrations, while particle adsorption and desorption behaviour dramatically changes. Therefore, it strongly supports that PEG adsorption onto silica surface and the oil–water interface might be helpful, but the depletion pressure is essential for better stability of Pickering emulsions.

### Criteria for achieving processable HIPPEs

The increased stability of a Pickering emulsion with hydrophilic silica particles by depletion attraction could form stable HIPPEs up to *φ*=0.9 even with a low concentration of the particles (Supplementary Figs 2, 4, 5), such as 0.54 vol% of silica particles, compared with the total HIPPE volume with the presence of 3.3 wt% PEG. This condition provides a stable HIPPE whose structures remain at least several weeks in the absence of any external force, although this amount of silica covers only about 6% of ∼40 μm diameter of oil droplet surface (Fig. 2a; see Methods).

However, such a low amount of particles could stabilize HIPPEs, such as a bridging particle monolayer mechanism^13^,^38^. Our micron-sized silica particles could form a relatively dense monolayer between two droplets, because of the capillary attraction to reduce menisci around the particles, thus decreasing the overall surface energy. Moreover, other stable Pickering emulsions with a low amount of micro-particles have been also reported as well^39^,^40^.

**Figure 3:**
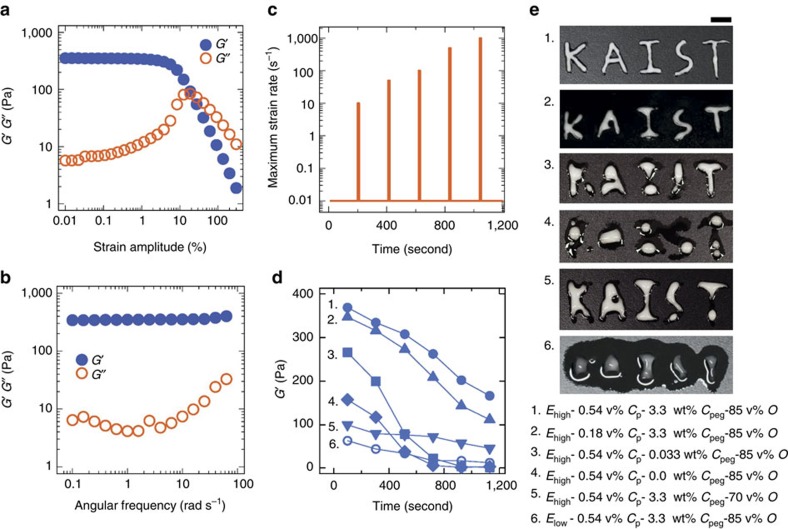
Rheology and processability of HIPPE. (**a**) Strain amplitude sweep and (**b**) frequency sweep for oscillatory complex moduli for HIPPE (0.54 vol% particles and 3.3 wt% PEG). (**c**,**d**) Linear shear elasticity measurements (blue, (**d**)) after application of intermittent high shear stress (orange, (**c**)). (**e**) Photographs of HIPPEs processed using the six samples in **d**. During the process of writing letters, the destabilization of HIPPE samples are observed. Images are taken 10 min after writing letters, and the shown structures are sustained at least several hours. *E*_high_, *E*_low_, *C*_p_, *C*_peg_ and *O* denote a high-energy emulsification, a low-energy emulsification, a particle concentration, a PEG concentration, and an oil fraction, respectively. Scale bar, 1 cm.

Once stable HIPPEs are obtained, we are able to process the HIPPEs, which have been very difficult to achieve^24^,^41^. Figure 3a–d; Supplementary Figs 11–14 show the linear (Fig. 3a,b; Supplementary Figs 11–13) and nonlinear (Fig. 3c,d; Supplementary Fig. 14) viscoelasticity of HIPPEs with various particle concentrations, as well as for various *φ*s, exhibiting a general signature of yield stress materials^10^,^11^. The elastic modulus of poly-disperse HIPPE is empirically expected to be (*G′*/(*σ*/*d*)=1.769*φ*^1/3^(*φ*−0.712)), where *σ* is the interfacial tension of oil/water, and *d* is the droplet size^11^. Our measurements and the averaged droplet size are in good agreement with this theory (Supplementary Fig. 11).

We found that two requirements should be satisfied to make HIPPE processable. First, large *G′* at small strains (linear regime) is required to minimize the deformation by external stresses. Second, at the same time, during typical processing accompanied by large shear stresses, an HIPPE must maintain its size *d* without coalescence, so that *G′*∼*σ*/*d* can be maintained^10^,^11^. To test this, we measure the linear elastic modulus (*G′*) after applying intermittent large shear strains (Fig. 3c,d; Supplementary Fig. 14). The HIPPEs formed with higher PEG concentrations at which sufficient depletion pressure existed show high enough elastic modulus values (>100 Pa), even after the application of five different large strain rates from 10 to 1,000 rad s^−1^. However, for HIPPEs formed with low PEG concentrations or without PEG, the elastic moduli rapidly approaches zero with increasing maximum strain rate, indicating the nearly complete collapse of the internal structure.

This high endurability against to the large external force could also support the significant role of the depletion pressure on the particle detachment from the oil–water interface as well. In Fig. 3d,e, two different HIPE samples (2 and 3) that contain the similar amount of particles at the oil droplet interface, but have different magnitude of depletion pressure, shows significantly different processability. Furthermore, more particles, higher energy input during emulsification and higher internal phase fraction enable the formation of more rigid and processable HIPEs, and these results agree well with our processing demonstration from various emulsion systems using a micropipette (Fig. 3e; Supplementary Movies 1 to 3).

### Universality and applicability of this strategy

**Figure 4:**
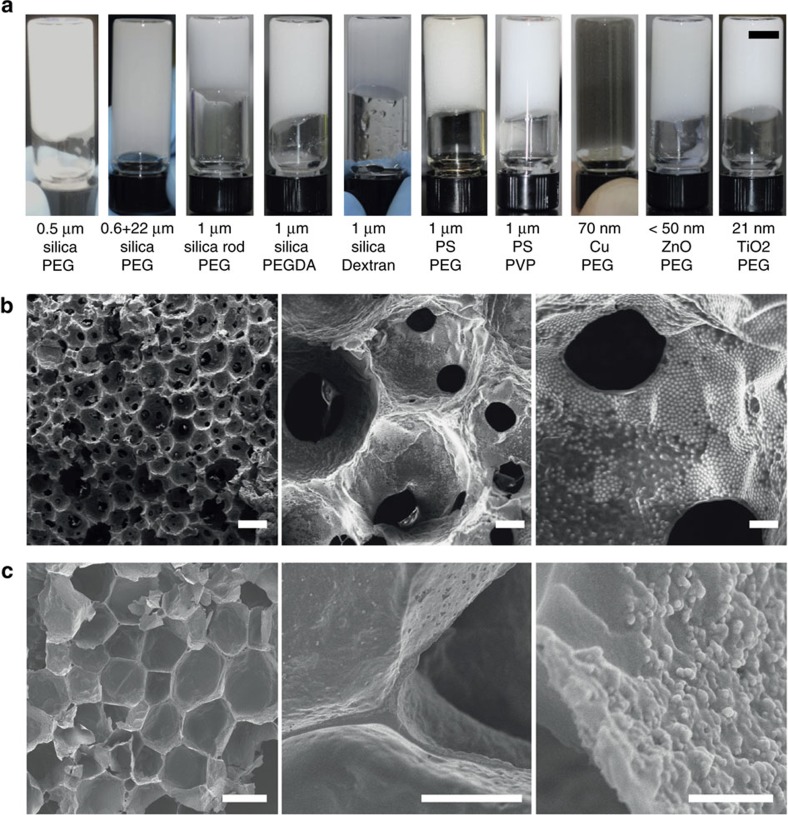
Various HIPPEs and synthesis of porous materials. (**a**) Various stable HIPPEs formed with various particles and depletants. Scale bar, 5 mm. (**b**,**c**) SEM micrographs of polymeric porous material. (**b**) Porous material produced by photo-crosslinking continuous phase and freeze-drying stable HIPPE that contained 0.54 vol% silica particles, 10 wt% PEG, and 10 wt% PEGDA. Scale bars, 100 μm (left), 20 μm (middle) and 5 μm (right). (**c**) Porous materials produced by the stable HIPPE that contains 0.3 vol% TiO_2_ particles, 26.5 vol% acrylic acid, 3 wt% PEGDA, and 1.5 wt% PEG. SEM micrographs indicate that both 1 μm-silica and 21 nm-TiO_2_ are well exposed at the surface of the internal phase. Scale bars, 100 μm (left), 20 μm (middle) and 500 nm (right).

Finally, we should emphasize that depletion attraction is a physical force that is independent of the surface chemistry of particles and the type of depletant employed. Therefore, this strategy would work for other systems, as long as the particles and depletant are well dispersed in a continuous phase such that no strong binding of the depletant to particle surfaces and oil/water interfaces occurs. Figure 4a demonstrates that various HIPPEs could be formed using metal, ceramic or polymeric particles. The size of these particles spans nearly three orders of magnitude (10 nm to a few μm), with various molecular weight PEGs (Supplementary Fig. 15), poly(ethylene glycol) diacrylate (PEGDA), and dextran as the depletant. These HIPPEs are stable for at least several days once formed, whereas stable HIPEs could not be formed without the help of the depletant. Furthermore, we demonstrate that a variety of porous polymeric materials could be produced from processable HIPPEs by crosslinking the continuous phase (Fig. 4b,c; Supplementary Figs 4 and 5). We clearly observe that the surfaces of these macro-porous materials is decorated with particles, whose density is even controllable by changing an initial amount of particles in the HIPPEs. (Supplementary Fig. 5).

## Discussion

We, for the first time, experimentally demonstrate that the depletion interaction could enhance the particle adsorption to the oil/water droplet interface and simultaneously suppress the particle desorption from the interface. This enhanced adsorption and suppressed desorption could stabilize emulsion even with highly hydrophilic silica particles. Based on these understandings, we propose a universal route to fabricate processable HIPPEs. Unlike previous researches^16^,^17^,^18^,^19^,^20^,^22^,^23^,^24^,^25^, each of which requires a specific strategy to form a stable HIPE, our technique works for particles with a wide range of size, shape and surface chemistry so long as they are dispersed in a continuous phase with diverse non- or weakly-adsorbing polymers as depletant. By evaporating a disperse phase and solidifying a continuous phase, (or by evaporating both phases and solidifying the interface) it is also possible to create various porous organic-inorganic hybrid materials with high porosity, the surface of which is covered with particles that could be post-functionalized. Our strategy can be applied to create more complicated and hierarchical structures by controlling the intra-structures of droplets or by the use of meso-/micro-porous particles, thus could enable unprecedented material designs for myriad applications such as absorbents, separation membranes, energy conversion/storage and other functional materials^42^,^43^,^44^,^45^,^46^,^47^.

## Methods

### Emulsion preparation

Before preparation of the emulsions, all particles are washed to remove any impurities (for example, surfactants) in the purchased particle solutions. All particles are first centrifuged, and then the supernatant aqueous solution is replaced with ethanol or deionized water. This washing process is repeated at least three times. To impart more hydrophilicity to the silica particles, the supernatant solution is sometimes replaced by HCl (37%, Sigma-Aldrich) and 1 M NaCl solution instead of DI water. This solution is then placed on a shelf for 24 h (for each variant of HCl and NaCl) and rinsed with DI water at least five times.

As shown in Supplementary Fig. 1, oil-in-water (O/W) emulsions is prepared by breaking up hexadecane (99%, Sigma Aldrich) in deionized water (>18 MΩ) that contained 1-μm silica particles (Polysciences, Inc., ±0.05 μm-s.d., 10% solid content in water), in the presence/absence of 10 k polyethylene glycol (Sigma Aldrich, Mn (number average molecular weight) 10,000 g mol^−1^) for 30–60 s. This is done using a vortex at 3,000 r.p.m. (Vortex mixer, DAIHAN Scientific) for low-energy emulsification and using a homogenizer at 30,000 r.p.m. (PT1300D, Kinematica) for high-energy emulsification, respectively. A portion (0.1 ml) of the silica particle solution (10 wt% in water), of which the particle fraction varies from 0 to 1.5 vol% (compared with total volume of oil and water), is first added to a water phase that contained 0–30 wt% of PEG (compared with the only water phase). Low or high-energy emulsification then proceeds with addition of the desired amounts of hexadecane. Depending on the volume fraction of the oil phase, which varied from 20 to 70%, each volume of oil and water is determined with a fixed total volume of 3 mL. High internal phase Pickering emulsions (HIPPE) is also prepared by additional emulsification steps from stable 70 vol% O/W emulsions by the sequential addition of oil. Starting from 1.5 ml total volume of 70 vol% O/W emulsions, the additional emulsification is performed by adding 0.75 ml of hexadecane to obtain 80 vol% HIPPE. One more emulsification step with the addition of 0.75 ml of hexadecane also results in an 85 vol% oil-in-water HIPE. Through this multi-emulsification process^25^, HIPPEs could be produced with various high internal oil fractions of 80, 85 or 90%.

### High internal phase Pickering emulsion preparation

To verify the universality of depletion-based HIPPE formation, various kinds of particles and depletants is used at 0.18–0.54 vol% (compared with the total volume of oil and water) particle fractions and 1–10 wt% (compared with the only water phase) depletant concentrations. In this work, 0.5 μm silica particles (Polysciences, Inc.), 22 μm+0.6 μm polydisperse silica particles that were wet ground in an attrition mill (700 r.p.m. for 4 h, Korea Material Development Co.) and purified by a filter paper (Whatman No. 541), 1 μm silica rods (0.3-μm diameter, 1-μm length, directly synthesized using a wet chemical method in an emulsion system^48^), 1 μm carboxylate polystyrene microspheres (Polysciences, Inc.), 70 nm copper nanoparticles (≥99.5%, Sigma-Aldrich), ≤50 nm zinc oxide (ZnO) nanoparticles (≥97%, Sigma-Aldrich) and 21 nm titanium dioxide (TiO_2_) nanoparticles (≥99.5%, Sigma-Aldrich) are used for HIPPEs with PEG as a depletant. Next, various pairs of particles-depletants are applied with 1 μm silica-PEGDA (polyethylene glycol diacrylate, Mn. 700, Sigma-Aldrich), 1 μm silica-dextran (Mr. (relative molar mass) 15,000–25,000, Sigma-Aldrich), and 1 μm PS-PVP (polyvinylpyrrolidone, Mw. (weight average molecular weight) 10,000, Sigma-Aldrich). Moreover, various molecular weight PEGs (Mn. 600, 3,000, 10,000, 350,000, 100,000) are used as depletants for the HIPPEs.

### Particle adsorption test

To investigate the effect of PEG concentration on the particle adsorption to the oil–water interface, a particle adsorption test is conducted at 22 °C. Before the experiment is conducted, the silica particle solution is washed with two different methods: (1) only DI water and (2) with HCl+NaCl, but they provides almost identical results. Then, 1 ml of hexadecane is added to 2.9 ml of deionized water that contains various concentrations of PEG (0.0–3.3 wt%, compared with the only water phase), and 0.1 ml of 10 wt% silica particle solution is added to the water phase. This oil–water mixture is then emulsified using both low- and high-energy protocols for 60 s and left on a shelf for 12 h. The particle fraction partitioned into the oil–water interface is measured by silica particle concentrations dissolved in the water phase. Here, ultraviolet–vis absorbance spectroscopy (UV 2600, Shimadzu Corp.) is used to measure the concentration of silica suspension at 900 nm. The calibration curve of absorbance of light as a function of silica concentration is first obtained. Then, the concentration of silica particles is calculated from the absorbance of light based on this calibration curve. Only the linear regime of the calibration curve is used to calculate silica concentration.

### PEG adsorption test

PEG adsorption to the oil–water interface is measured by the pendent drop technique at 22 °C. Water droplets of 25 μl that contains various PEG concentrations from 0 to 3.3 wt% (compared with the only water phase) are first introduced at the end of a glass syringe (0.75 mm diameter, Hamilton) in the bulk oil phase. Images are then recorded of each droplet using a CCD camera (WAT-902H, Watec), and the shapes of the droplets are analysed to extract information about the interfacial tension. More details are explained by Stauffer *et al*.^49^.

PEG adsorption onto the silica surface is investigated by the molybdophosphoric acid method at 22 °C (ref. 50). Molybdophosphoric acid reagent is first made by dissolving 0.5 g of molybdophophoric acid hydrate (ACS reagent, Sigma-Aldrich) and 0.5 g of barium chloride dihydrate (99%, Sigma Aldrich) in 250 ml of DI-water. Then, 1.5 ml of hydrochloric acid (37%, Sigma Aldrich) is further added to this solution. Next, PEG solutions in DI-water with various concentrations is vortexed at 3,000 r.p.m. with the particle solution that is washed by ethanol and DI water before the experiments is conducted, and this mixture is then centrifuged at 14,000 r.p.m. for 10 min to collect the supernatant PEG solution. The reagent solution is added to the supernatant PEG solution, and this is shaken gently to precipitate the PEG-molybdophosphoric acid complex. Then, the mixed solution with the precipitate is centrifuged at 14,000 r.p.m. for 10 min, and a supernatant solution is obtained again. Using ultraviolet–vis absorbance spectroscopy, the remaining amount of molybdophosphoric acid reagent in the supernatant solution is measured at 215 nm, and the amount of PEG that is reacted with the reagent is calculated. Finally, the amount of PEG that is attached to the silica surface could be calculated by subtracting the amount of PEG that has reacted with the reagent from the initial concentration of PEG solution in the second stage. This result agrees well with the previous result^51^.

### Particle desorption test

A particle desorption test is conducted at the air-water interface due to the technical difficulty of doing so at the oil/water interface. A Langmuir trough (KSV NIMA) is first filled with various concentrations of PEG solution (from 0 to 3.3 wt%, compared with the only water phase), and 400 μl of 10 wt% silica particle in ethanol solution is spread with a micro-syringe onto a clean air-water interface. Roughly 15 min is then allowed for evaporation of the solvent, before compression of the particle monolayer. During a full cycle of compression–expansion (20–200 cm^2^ of surface area), the surface pressure at the air–water interface is measured using a Wilhelmy plate (KSV NIMA). Each experiment is conducted for three cycles of compression-expansion with a barrier speed of 0.5 cm min^−1^ at 22 °C.

### Particle coverage at the oil/water interface

The particle amount for covering the whole surface of droplets is estimated from the method proposed by Wiley *et al*.^52^. For instance, if there are *N* oil droplets with an average diameter, *D*∼40 μm (Supplementary Fig. 11), in 3 ml of a total HIPPE volume, the total oil volume (*V*_oil_) with 0.85-volume fraction is *NπD*^3^/6, resulting in *N*=6*V*_oil_/*πD*^3^. The total area of an oil–water interface (*A*), is, thus, *D*^2^=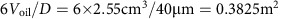. We assume that *n* colloid particles of diameter *d* are 2d cubic closed packed at the total interfacial area, *A*=*nd*^2^=0.3825, m^2^, thus indicating that 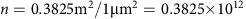. Therefore, the volume fraction of particles requires to cover the whole interfacial area is ∼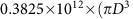/6)/3 cm^3^ ∼6.67 vol%, compared with the total HIPPE volume.

### Linear and non-linear rheology

Before the rheological properties of each emulsion system is identified using a rheometer (MCR 302, Anton Paar) with parallel plate geometry (1 mm gap, 25 mm diameter), various O/W emulsions from 50 to 85% oil fractions are prepared with various fractions of silica particles (from 0.18 to 0.54 vol%, compared with the total volume of oil and water) by low- and high-energy emulsifications. After the linear regime is checked by strain sweeping of various emulsion samples, linear oscillatory rheology is first performed with frequencies ranging from 0.1 to 100 rad s^−1^ at a fixed strain amplitude (0.1%). The shear moduli at a fixed frequency (10 rad s^−1^) are then measured with various strain amplitudes ranging from 0.01 to 300%.

Non-linear rheology experiments are also conducted with a strain amplitude range of 100–2,000% and with 10–50 frequencies to investigate the structural resistance of the materials to the external stress. The rheological properties of the HIPPEs and emulsions are first measured for 200 s with a linear regime amplitude of 0.1% and a frequency of 10 rad s^−1^, followed by much higher shear strain amplitudes and frequencies for 10 s that are able to destroy the emulsion microstructures. Immediately after the application of high shear stress, the same small amplitude and frequency (0.1%, 10 rad s^−1^) are applied again for 200 s to measure the linear rheological properties of the emulsion system. This process is repeated with gradually increasing frequency and amplitude (100%, 10 rad s^−1^; 500%, 10 rad s^−1^; 1,000%, 10 rad s^−1^; 1,000%, 50 rad s^−1^; and 2,000%, 50 rad s^−1^). All rheology experiments are performed at 25 °C.

### Data availability

The data that support the findings of this study are available from the corresponding author on reasonable request.

## Additional information

**How to cite this article**: Kim, K. *et al*. Processable high internal phase Pickering emulsions using depletion attraction. *Nat. Commun.* **8**, 14305 doi: 10.1038/ncomms14305 (2017).

**Publisher's note**: Springer Nature remains neutral with regard to jurisdictional claims in published maps and institutional affiliations.

## Supplementary Material

Supplementary Information Supplementary Figures 1-15, Supplementary Methods and Supplementary References

Supplementary Movie 1 Lettering of HIPPE (0.54 v%-silica particle, 3.3 wt%-PEG) by a micropipette (10-200 μl, Eppendorf).

Supplementary Movie 2 HIPPE stream (0.54 v%-silica particle, 3.3 wt%-PEG) in an oil phase (hexadecane) by a micropipette (10-200 μl, Eppendorf).

Supplementary Movie 3 HIPPE stream HIPPE stream (0.54 v%-silica particle, 3.3 wt%-PEG) in a water phase by a micropipette (10-200 μl, Eppendorf).
