# Supplementary material for: Processable high internal phase Pickering emulsions using depletion attraction
Source: Nat Commun. 2017 Feb 1;8:14305. doi: 10.1038/ncomms14305 (PMC5296636; doi:10.1038/ncomms14305)
Supplement: Supplementary Information — Supplementary Figures 1-15, Supplementary Methods and Supplementary References [file ncomms14305-s1.pdf]

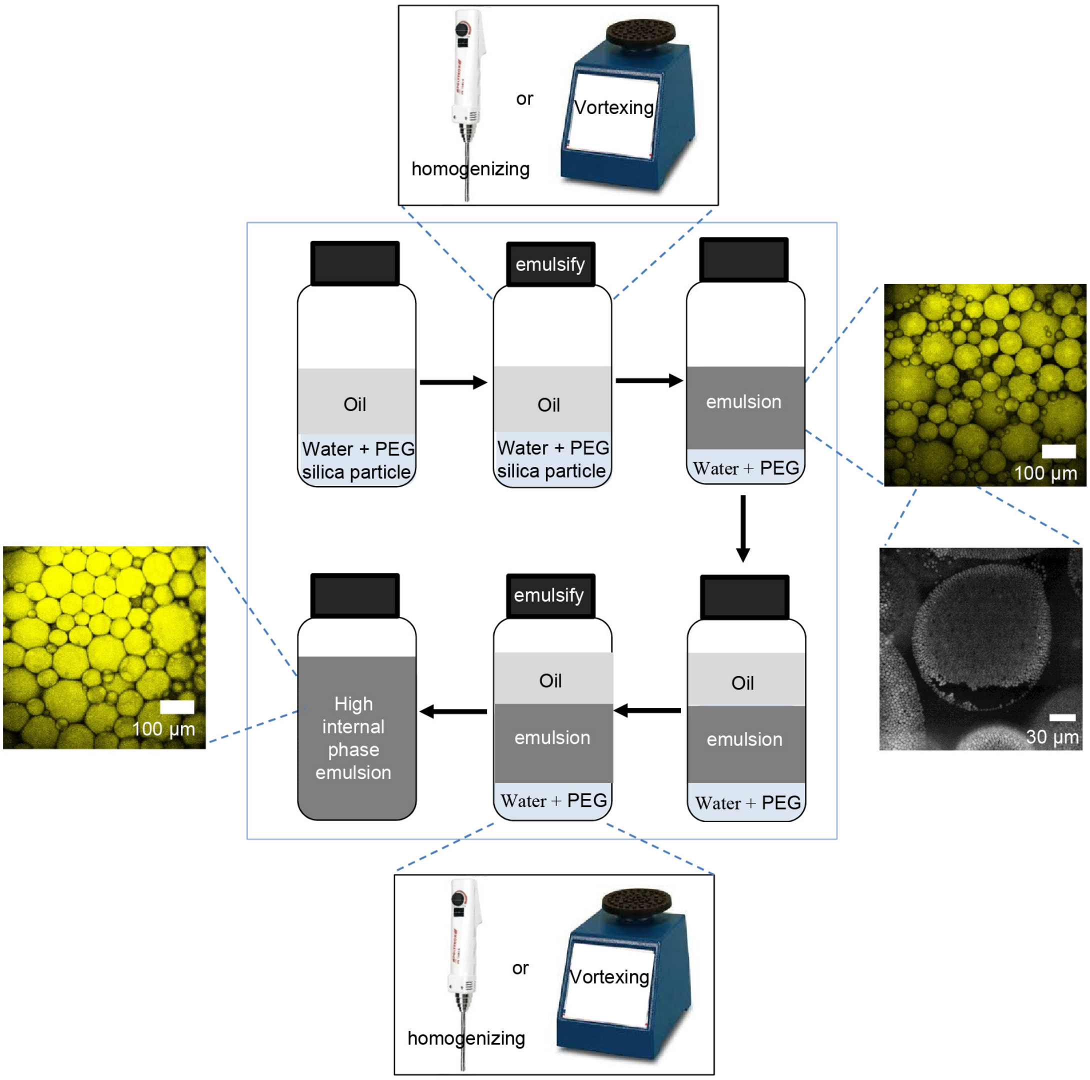


**Supplementary Figure 1. Simple schematics for preparing oil-in-water (with PEG) emulsions with silica particles by low or high energy emulsifications.**

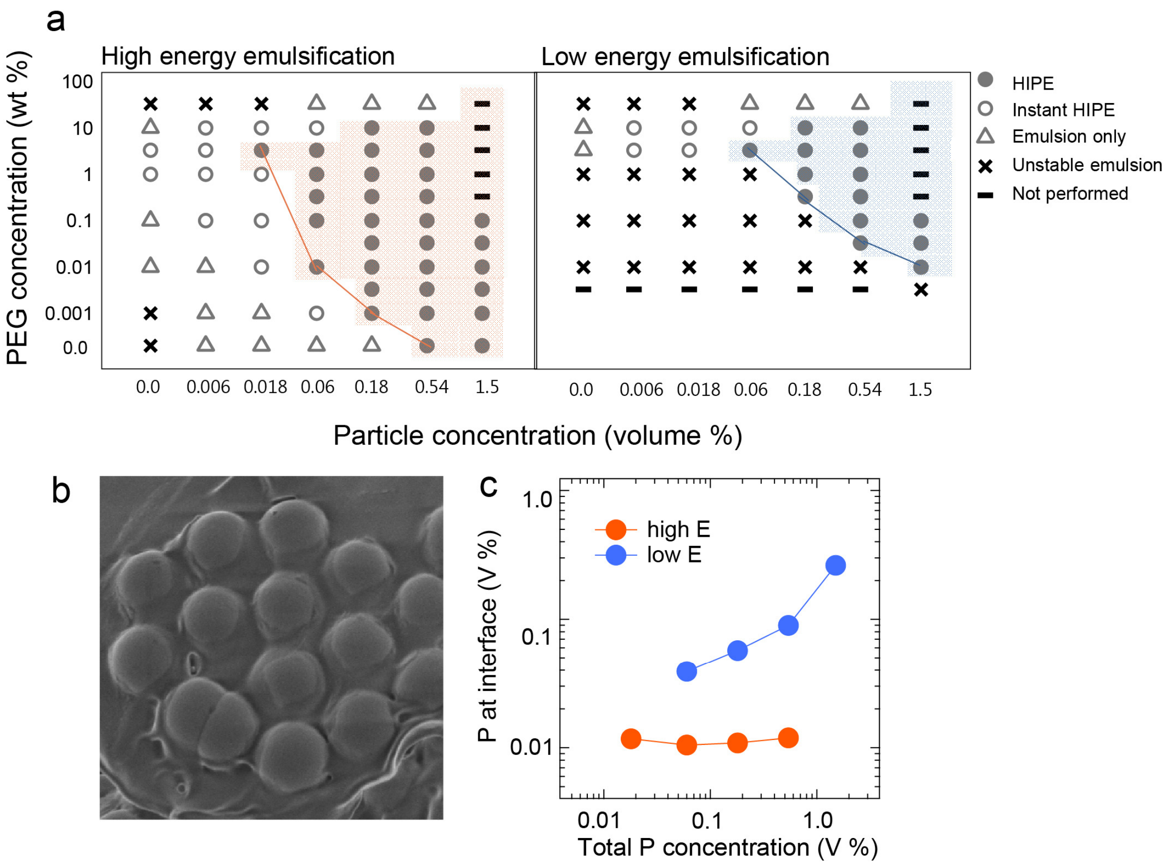


**Supplementary Figure 2. Phase diagram for stable HIPPE formation and its criteria. (a)** The stabilization of HIPPEs that contain various concentrations of PEG and silica particles are performed with two mixing methods: high-speed mixing with a homogenizer for high-energy emulsification and simple vortexing for low-energy emulsification. (**b**) SEM micrographs indicate that the particles are trapped at the oil/water interface[2], exposing a part of the particle surface to an oil phase with a contact angle of ~ 0°. (**c**) Stable HIPE seems to require a minimum particle concentration at the oil-water interface. In the case of the high-energy method, this value is almost constant (~ 0.012 v%) even with varying particle concentrations applied initially in the aqueous bulk. A greater minimum amount of particle at the interface is required to achieve stable HIPPEs with increasing particle concentration in the bulk when low-energy emulsification is applied.

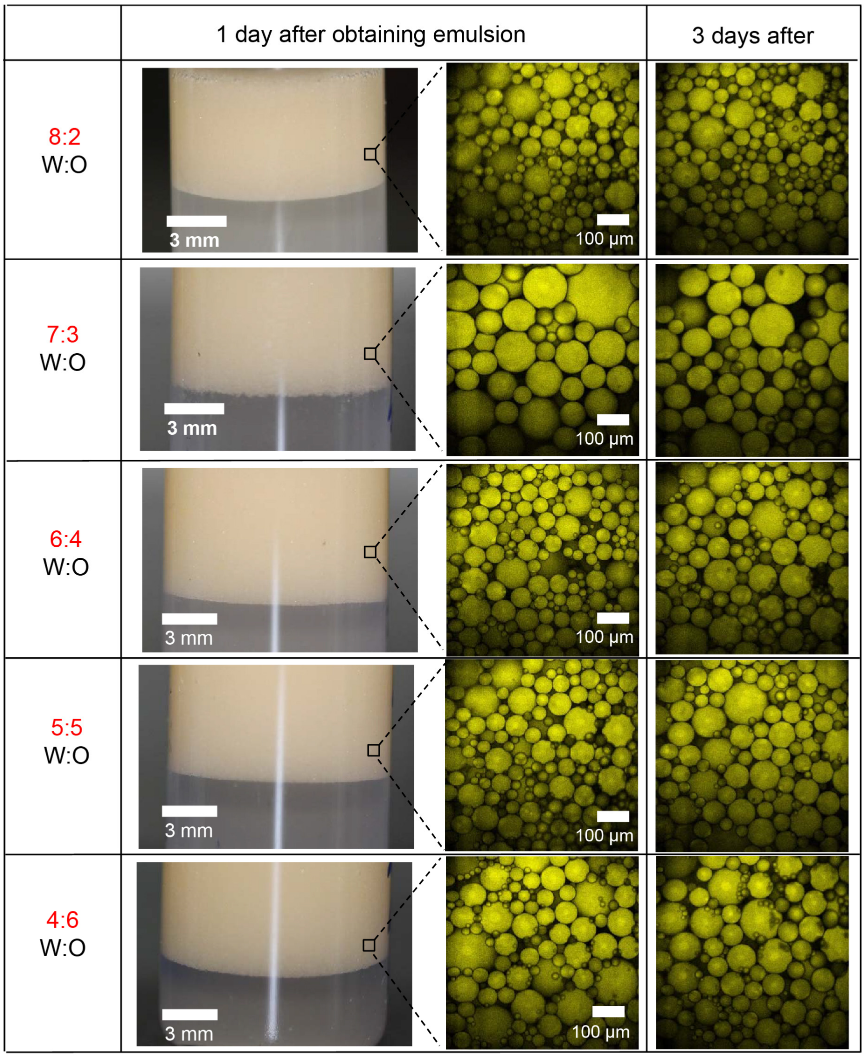


**Supplementary Figure 3. Macroscopic and microscopic images of emulsions containing 0.18 v% of 1 μm silica microspheres with 1 wt% of PEG.** Even though the oil fraction, $\phi$, varied from 0.2 to 0.6, the creaming process occurs in all samples after low-energy emulsification, thus, demonstrating that oil droplets are concentrated with an excess water phase. Observing oil droplets in all emulsions, we find that they show similar size distribution even with various initial oil fractions. Furthermore, all emulsions show little difference between structures that are equilibrated for 1 day and for 3 days, respectively, thus confirming that the structure of emulsions is in a pseudo-equilibrium state within a day time-scale. For the visualization of oil droplets, an oil phase is dyed with 5 μg/ml of Nile red.

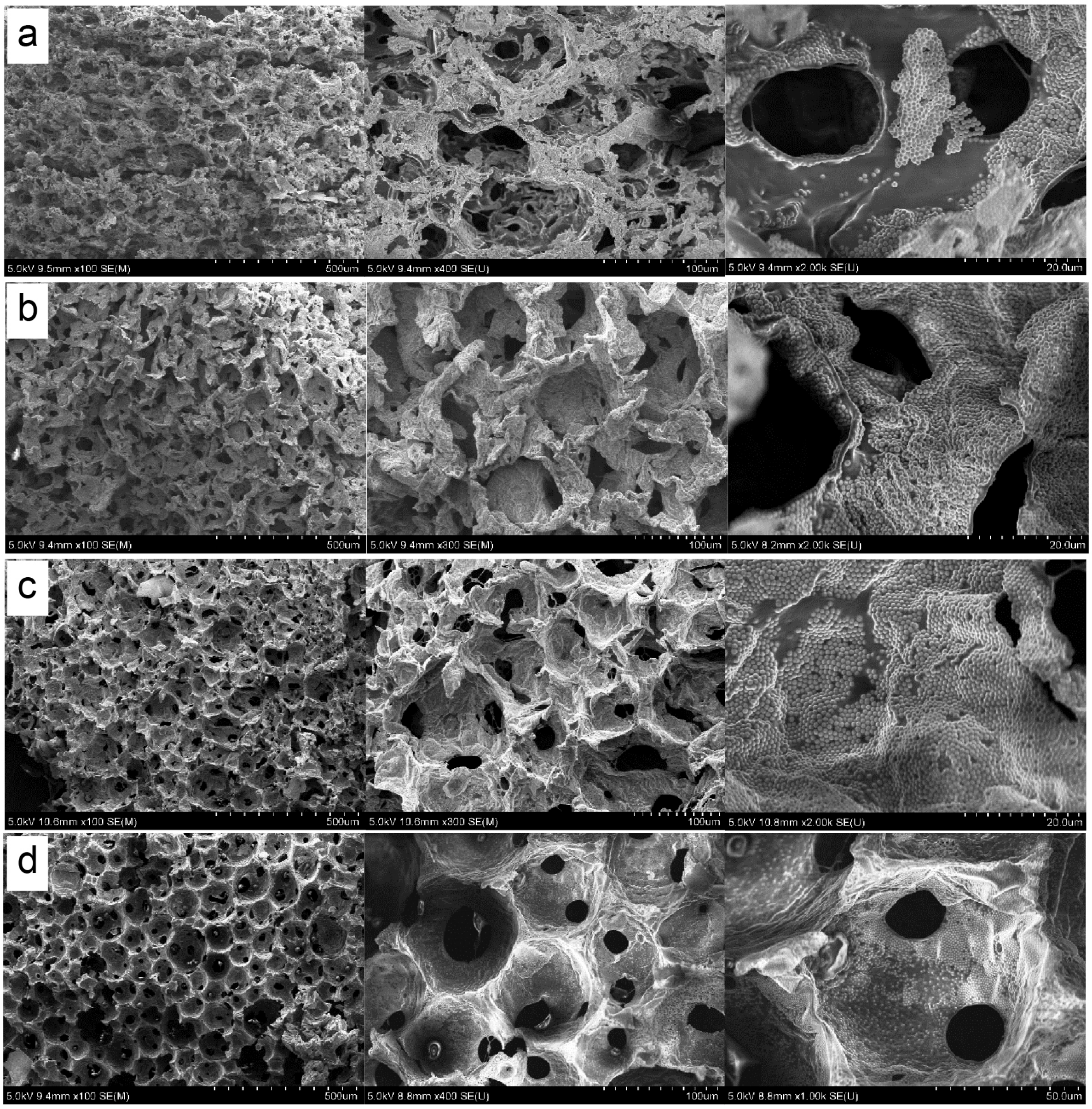


**Supplementary Figure 4. SEM images of porous materials from HIPPEs with various PEG concentrations.** Using low-energy emulsification with 0.54 v% of 1 μm-silica particles, HIPPEs are first produced with both PEGDA and PEG. Here, a fixed amount of PEGDA (10 wt%) is used with a photo-initiator (15 wt% to PEGDA), while the concentration of PEG is varied (**a.** 0.0 wt%, **b.** 0.1wt%, **c.** 1 wt%, **d.** 10 wt%.). After emulsification, UV light is applied to the HIPPEs to solidify the continuous phase by crosslinking PEGDAs, and these systems are then freeze-dried for 24 hours, prior to SEM imaging.

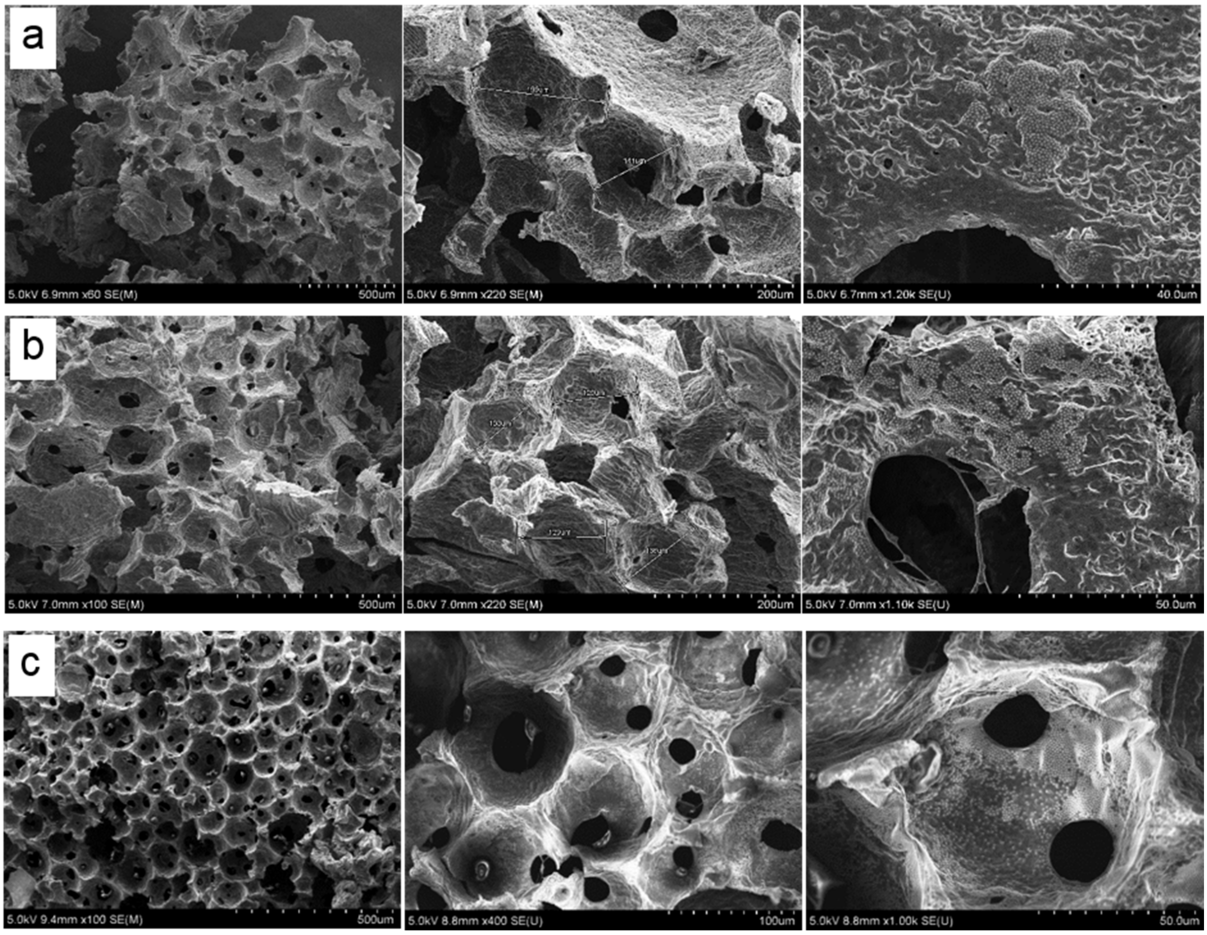


**Supplementary Figure 5. SEM images of porous materials from HIPPEs with various particle concentrations.** Using low-energy emulsification, HIPPEs are produced with fixed amounts of PEGDA (10 wt%), PEG (10 wt%), and a photo-initiator (15 wt% to PEGDA) while the concentration of 1μm-silica particles is varied (**a.** 0.06 v%, **b.** 0.18 v%, **c.** 0.54 v%). After emulsification, UV light is applied to the HIPPEs to solidify the continuous phase by crosslinking PEGDAs, and these systems are then freeze-dried for 24 hours, prior to SEM imaging.

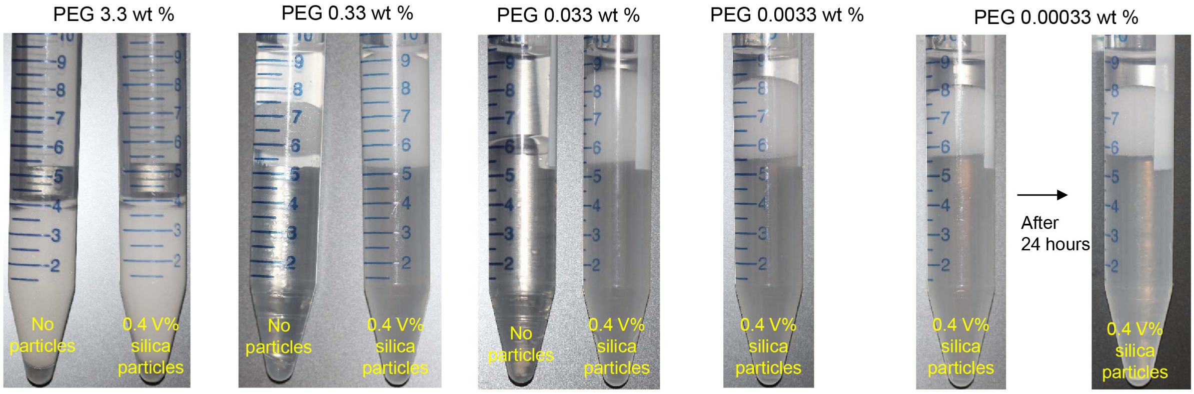


**Supplementary Figure 6. Emulsion destabilization by reducing the PEG concentration from 3.3 to 0.00033 wt%.** With the presence/absence of 0.4 v% of 1 μm-silica particles, emulsions (70% oil fraction) are produced with 3.3 wt% of PEG. The concentration of 3.3 wt% PEG in a water phase is then reduced to 0.33 wt% by the addition of fresh DI water. By removal of the PEG solution (0.33 wt%) and the addition of fresh water again, the concentration of PEG reaches 0.033 wt%, thereby enabling the emulsion with 0.4 v% of particles to remain stable, while the emulsions without particles destabilize completely. These processes are repeated twice more to obtain a 0.00033 wt% concentration of PEG, and this emulsion still survives even after 24 hours. This delayed destabilization by irreversible adsorption is likely due to contact line pinning at the rough silica surface[2].

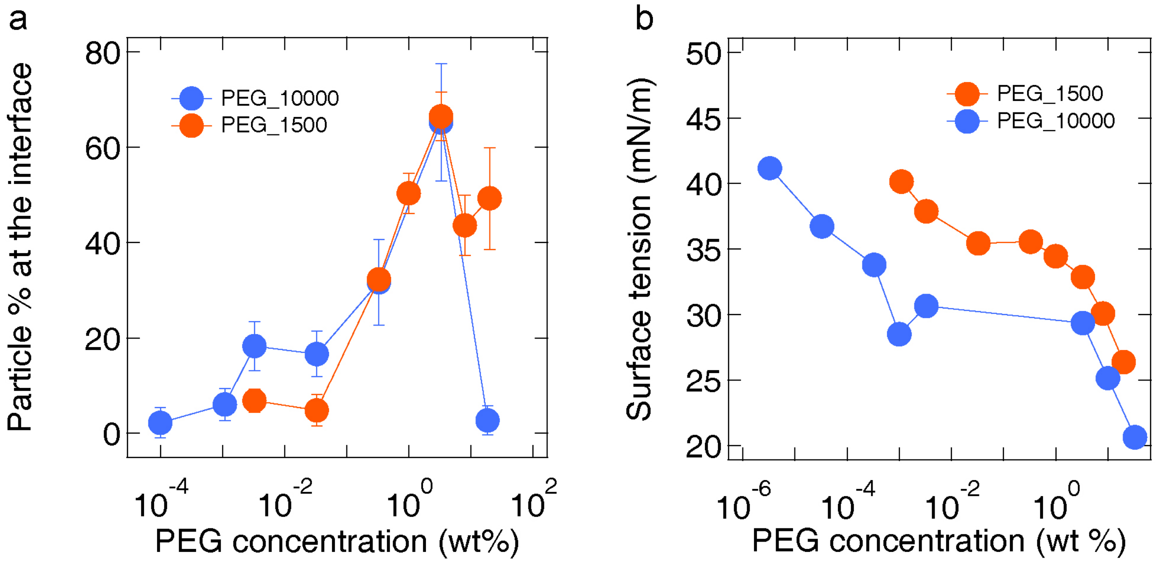


**Supplementary Figure 7. A comparison between PEG-10000 and PEG-1500.** (**a**) The particle adsorption behavior with PEG concentration. Error bars denote standard deviation of the value estimated in three different experiments. (**b**) The interfacial tension of oil/water, measured 5 minute after loading the sample in a pendent drop method. Here, three interesting features are observed in PEG-1500, such as a significantly lower adsorption of particles to the interface at the regime 1 (0.0033 wt% $\leq C_{peg} \leq$ 0.033 wt%), an almost similar adsorption of particles at the regime 2 (0.033 wt% $\leq C_{peg} \leq$ 3.3 wt%), and a significantly higher adsorption of particles at $C_{peg} >$ 3.3 wt%, compared to the results from the PEG-10000.

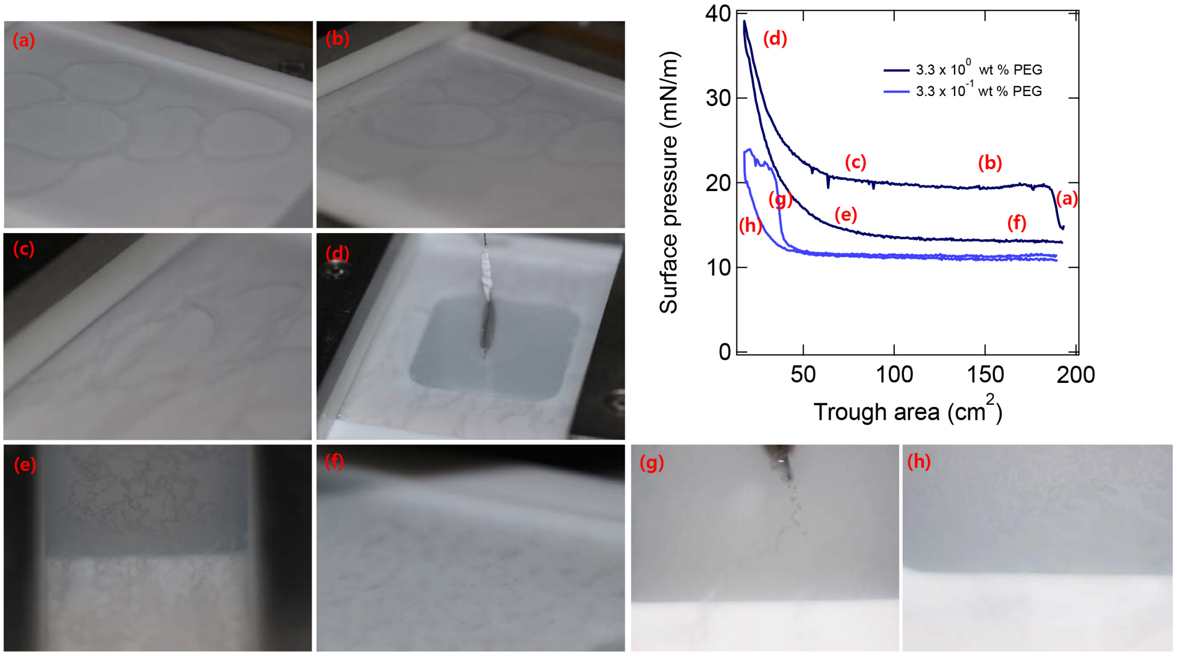


**Supplementary Figure 8. Images of 1 µm-silica particles at the air-PEG aqueous solution interface.** Macro-structures of 1 µm-silica particles at the air-water interface created in a Langmuir trough are observed during the first compression-expansion cycle with 3.3 and 0.33 wt% of PEG aqueous solutions.

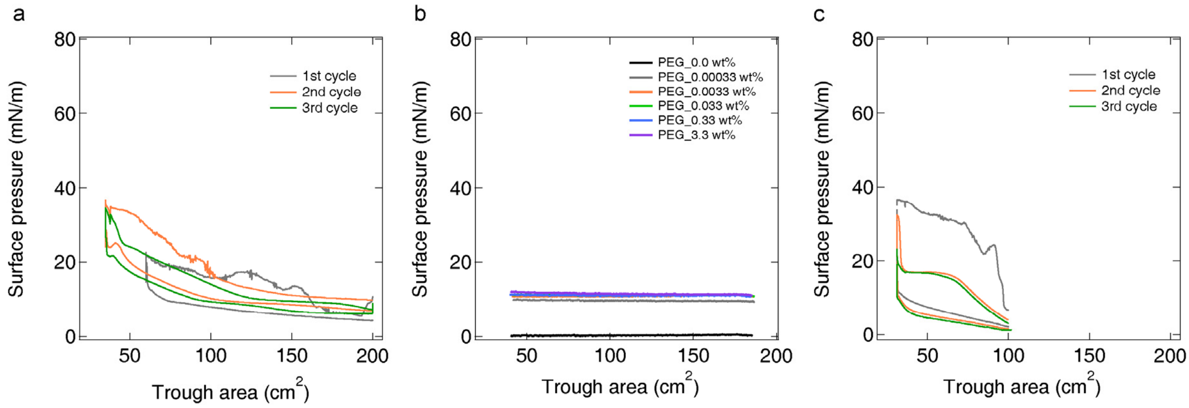


**Supplementary Figure 9. Various isotherms at the air-water interface.** (**a**) 150 μL of 10 wt% particle suspension (dispersed in pure ethanol) is spread on 200 $cm^2$ of the air/10 wt% dextran solution initially, and three compression-expansion cycles are then conducted during an entire experiment. Compression rate is 0.3 $cm^2$ / sec. (**b**) Surface pressure measurements of different PEG concentrations in the bulk at the air/water interface. While PEG concentration changes 3 orders of magnitude from 0.0033 wt% to 3.3 wt%, their surface concentrations are almost identical. (**c**) 2000 μL of 10 wt% particle suspension (dispersed in pure ethanol) is spread on 100 $cm^2$ of the air/water interface, initially. Three compression-expansion cycles are then conducted during an entire experiment. A compression rate is 0.3 $cm^2$ / sec.

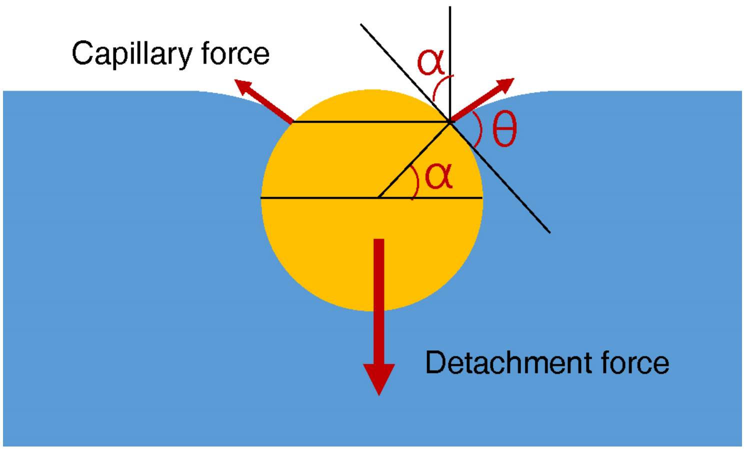


**Supplementary Figure 10. Maximum detachment force balanced with capillary force.** During the force calculation for the particle detachment, it is impossible to determine the contact angle, θ that determines the magnitude of the detachment force, but a maximum magnitude of the force could be simply estimated. Here, depletion pressure, $P_{de}$ in 3.3 wt % is ~ $100\,kPa$ and interfacial tension between oil-water with 3.3 wt % PEG, γ, is ~ 30 mN/m, as indicated in Fig. 2c. Thus, the magnitude of the detachment force that is balanced with the capillary force is ~ $\pi D * \cos\alpha * \gamma * \cos(\pi - \alpha - \theta)$[3,4]. Therefore, the maximum magnitude of the detachment force is ~ $\pi D * \gamma$ ~ 100 nN, whereas the magnitude of depletion force is ~ $\pi D^2\, P_{de}$ ~ $3.14 * 1\,(\mu m)^2 * 100\,kPa$ ~ 300 nN.

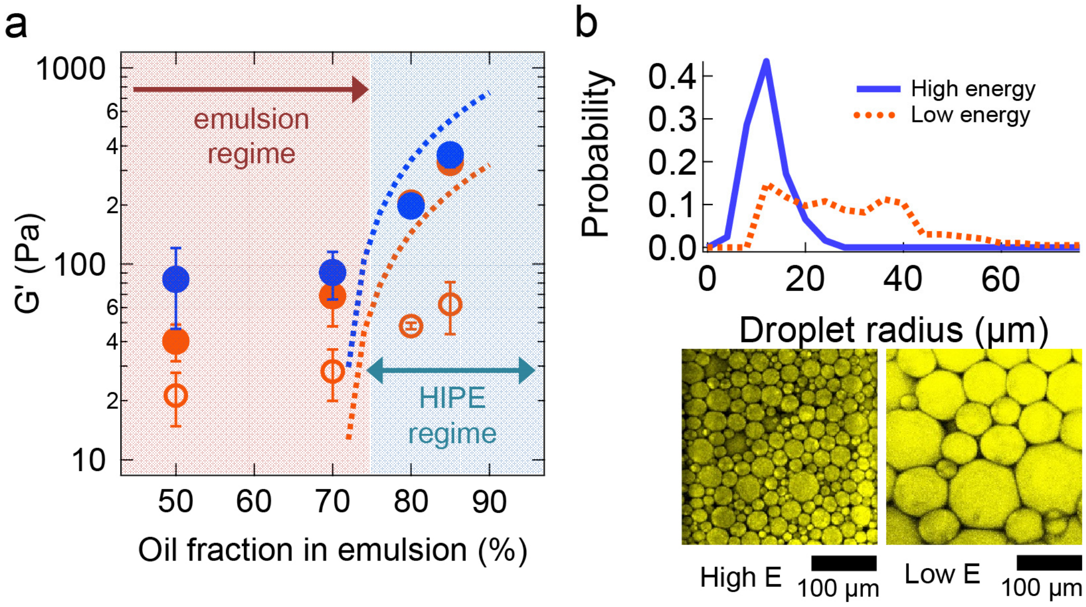


**Supplementary Figure 11. Linear rheology of HIPPEs with varying particle fraction.** (**a**) Elastic moduli as a function of the oil fraction in emulsion prepared by high-energy emulsification (solid circles) and low-energy emulsification (empty circle) and with various particle volume fractions (blue: 0.54 v%; orange: 0.18 v%). The dotted lines represent the Princen theory for polydisperse high internal phase emulsions[5]. Error bars denote standard deviation of the value estimated in three different experiments. (**b**) The size distribution of >200 droplets measured using confocal microscope images of both high- and low-energy emulsification.

.

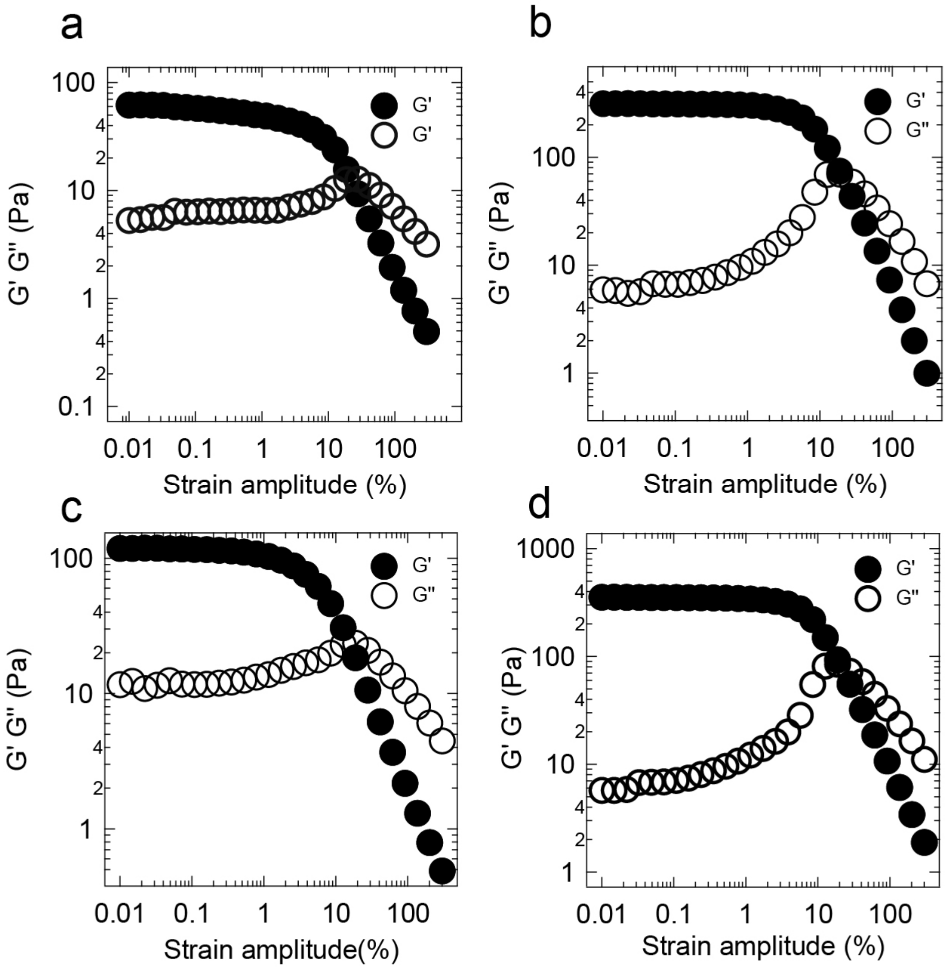


**Supplementary Figure 12. Oscillatory shear measurement of HIPPEs and emulsions with varying shear amplitude from 0.01 % to 300% at a fixed frequency of 10 rad/sec**. HIPPEs and emulsions are prepared by three different methods; low-energy emulsifications with 0.18 v% of particles, (**a.** 85 % of oil contents), high-energy emulsifications with 0.18 v% of particles (**b.** 85 % of oil contents), and high-energy emulsifications with 0.54 v% of particles (**c.** 70% and **d.** 85 % of oil contents). Even though the oil fraction in the emulsion system and the emulsification protocol are varied, universal similarity of the rheological properties of the emulsions and HIPPEs is observed here. For example, G' is larger than G'' at a relatively low shear amplitude regime, but G'' starts to dominate at high amplitudes. In addition, the decaying slope of G' is almost two times larger than that of G'' for a high amplitude regime and at a cross-over amplitude (~ 20 %), A pronounced peak of G'' is observed too. These observations indicate that HIPPES and emulsions are gel-like soft materials that have yield stress[6].

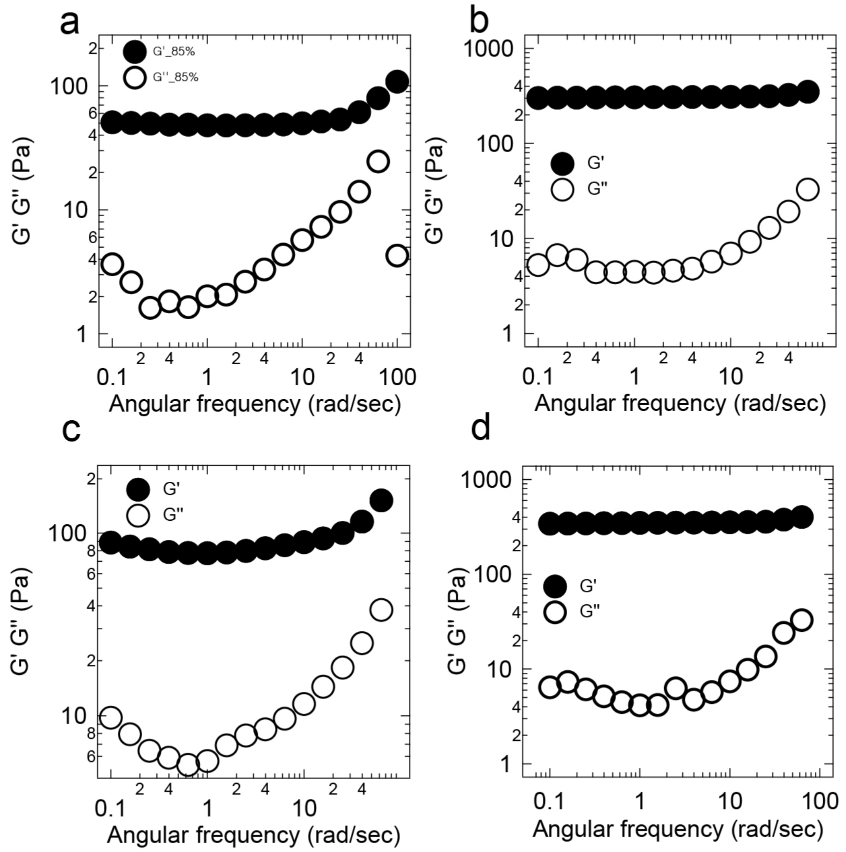


**Supplementary Figure 13. Oscillatory shear measurement of HIPPEs and emulsions with varying frequencies from 0.1 to 100 Hz at fixed strain amplitude of 0.1 %.** HIPPEs and emulsions are prepared in three different ways: low-energy emulsification with 0.18 v% of particles (**a.** 85 % of oil contents), high-energy emulsification with 0.18 v% of particles (**b.** 85 % of oil contents), and high-energy emulsification with 0.54 v% of particles (**c.** 70% and **d.** 85 % of oil contents). In this rheological experiment, ubiquitous similarity is also observed. G' shows weak dependence on frequency (ω), and G'' increases with ~ $\omega^{0.5}$ dependence, especially for a high-frequency regime. This universality also indicates that HIPPEs and emulsions are gel-like soft materials[6].

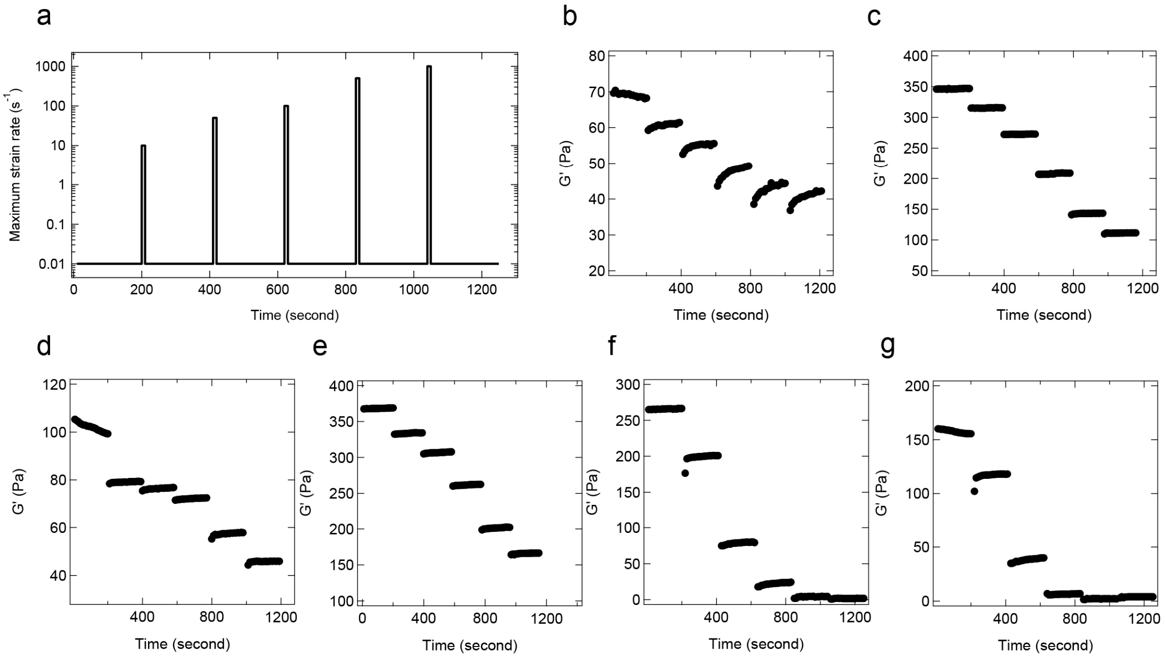


**Supplementary Figure 14. Oscillatory shear measurement of HIPPEs and emulsions before and after application of high shear amplitudes and frequencies.** (**a**) Various strain rates ($\gamma$•ω) with time during an oscillatory shear measurement. Rheological properties of HIPPEs and emulsions are measured for 200 s with 0.1% - $\gamma$ and 10 rad/sec - ω. A high shear amplitude and frequency are then applied for 10 s to destroy the emulsion structures, and the same 0.1% - $\gamma$ and 10 rad/sec - ω are applied again for 200 s to measure the rheological properties of the emulsion system after destruction. This process is repeated with gradually increasing frequency and amplitude (100%, 10 rad/sec; 500%, 10 rad/sec; 1000%, 10 rad/sec; 1000%, 50 rad/sec; and 2000%, 50 rad/sec). As a result, this linear-nonlinear rheological measurement is used to measure the mechanical properties of several different HIPPEs and emulsions that are prepared by (**b**) 85% oil fraction – 0.18 v% particle – low-energy emulsification – 3.3 wt% PEG, (**c**) 85% oil fraction – 0.18 v% particle – high-energy emulsification – 3.3 wt% PEG, (**d**) 70% oil fraction – 0.54 v% particle – high-energy emulsification – 3.3 wt% PEG, (**e**) 85% oil fraction – 0.54 v% particle – high-energy emulsification – 3.3 wt% PEG, (**f**) 85% oil fraction – 0.54 v% particle – high-energy emulsification – 0.033 wt% PEG, and (**g**) 85% oil fraction – 0.54 v% particle – high-energy emulsification – 0.0 wt% PEG.

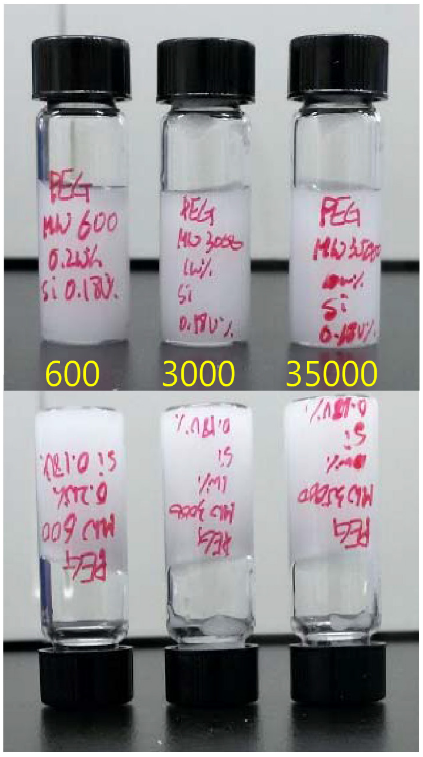


**Supplementary Figure 15. HIPPE formation with various PEG M.W.s** Various HIPPEs are emulsified with various molecular weights of PEG: 600, 3000, 10000, 35000, and 100000 g/mol. At a fixed number density of PEGs, 0.01 M, all HIPPEs are produced successfully, except for 0.01 M of 100k Da PEG.

## Supplementary Methods

### Confocal imaging for visualization of internal structure

Nile red molecules (≥ 98.0%, Sigma Aldrich) dissolved in hexadecane (5 μg/mL) are used for visualization of the oil droplets (Fig 1, c and d). To excite the Nile red molecules, a 488-nm laser (Coherent) is applied, and the emitted light is captured to visualize the microscopic structures using a customized confocal microscopy system[1]. It has a 75 μm-diameter pinhole and an extra-long working distance with a 50× objective lens with 0.5 N.A. (Olympus). Micrographs of 3 μm fluorescence silica particles (Biotech GmbH & Co. KG, excitation: 569 nm, emission: 585 nm), shown in Fig. 1e, are also visualized by the same confocal microscopy system, but with a different excitation laser (594 nm, Coherent).

### Droplet size in emulsions

To measure the average droplet size, two HIPPEs with 0.18 v% of 1-μm diameter silica microspheres and 1 wt% PEG are prepared by low- and high-energy emulsification, respectively. Confocal micrographs of the HIPPEs are then taken and converted to binary images in only black (oil droplets) and white (continuous phase). A size-range of 4 μm for histograms and a drop population of ~200 are used to establish the size-distribution curve of both emulsions produced by high- and low-energy emulsification. All image processing and analysis are conducted using *Image J* software (NIH).

### Scanning electron microscopy (SEM)

Internal structures of HIPPEs are investigated by scanning electron microscopy (SEM, S-4800, Hitachi). In order to obtain SEM images, HIPPEs samples are first prepared using 0.06-0.54 v% of silica (or $TiO_2$) particles (in total volume of emulsion), 0–10 wt% of PEG (in a water phase), 3-10 wt% of PEGDA (in a water phase) and 0-26.5 v% of acrylic acid (in a water phase, Sigma-Aldrich) with photo-initiator (15 wt% to PEGDA, 2-Hydroxy-2-methylpropiophenone, Sigma-Aldrich). Here, the addition of PEG is to keep the depletion pressure because PEGDA are consumed by the crosslinking. Then a UV light (Xcite-120Q, Lumen dynamics) is shined to the emulsion for 1 h to crosslink the continuous phase. Next, the prepared HIPPEs are freeze-dried (FDU-1200, EYELA) for 14 h, and these well dried samples are then loaded onto a carbon tape. Finally, the microstructures of the HIPPEs are visualized using SEM at a relatively low voltage (2–5 kV). To visualize the material surface in which $TiO_2$ particles are adsorbed, 8 nm thick platinum thin film is deposited on the material surface.

### Phase diagram

Our emulsions are classified into various kinds depending on their stability. First, ‘emulsion only’ indicates that a stable emulsion could be formed only with oil fractions up to 70%, but the addition of more oil (emulsification of 80% oil fraction) immediately destabilizes the emulsion. ‘Unstable emulsion’ denotes when even emulsions with 70% oil fraction are not stable. ‘Instant HIPE’ and ‘HIPE’ refers to HIPPEs that could only survive for

a few hours with a gradual destabilization and are stable for at least several days, respectively. For the phase diagram of stable HIPPEs, 1-μm silica microspheres and 10k Da PEG are used.

## Supplementary References


1. Kim, J. B. *et al.* Intravascular optical imaging of high-risk plaques in vivo by targeting macrophage mannose receptors. *Sci. Rep.* **6,** 22608 (2016).
2. Kaz, D. M., McGorty, R., Mani, M., Brenner, M. P. & Manoharan, V. N. Physical ageing of the contact line on colloidal particles at liquid interfaces. *Nat. Mater.* **11,** 138–42 (2012).
3. Nutt, C. W. Froth flotation: The adhesion of solid particles to flat interfaces and bubbles. *Chem. Eng. Sci.* **12,** 133–141 (1960).
4. Ally, J., Kappl, M., Butt, H.-J. & Amirfazli, A. Detachment Force of Particles from Air−Liquid Interfaces of Films and Bubbles. *Langmuir* **26,** 18135–18143 (2010).
5. Princen, H. . & Kiss, A. . Rheology of foams and highly concentrated emulsions. *J. Colloid Interface Sci.* **112,** 427–437 (1986).
6. Wyss, H. M. *et al.* Strain-rate frequency superposition: a rheological probe of structural relaxation in soft materials. *Phys. Rev. Lett.* **98,** 238303 (2007).
